# Notational Analysis of the Final Matches of the 2023 IBA Women’s World Boxing Championships

**DOI:** 10.3390/jfmk10030350

**Published:** 2025-09-12

**Authors:** Francesca Martusciello, Andrea Perazzetti, Arben Kaçurri, Marco Consolati, Antonio Tessitore

**Affiliations:** 1 Department of Movement, Human and Health Sciences, University of Rome “Foro Italico”, 00135 Rome, Italy; antonio.tessitore@uniroma4.it; 2 National PhD Programme in Kinesiology and Sport Sciences, Department of Neurosciences, Biomedicine and Movement, University of Verona, 37129 Verona, Italy; 3 Sports Research Institute, Sports University of Tirana, 1001 Tirana, Albania; akacurri@ust.edu.al; 4 National Teams Technical Commission, Italian Boxing Federation (FPI), 00196 Rome, Italy; marco.consolati@fpi.it

**Keywords:** female boxing, notational analysis, offensive effectiveness, defensive strategies, combat sports, performance analysis, elite athletes

## Abstract

**Background:** Recently, elite women’s boxing has undergone significant structural and regulatory changes, including the adoption of the 3 × 3-min-bout format and expanded Olympic categories. Despite increased female participation, scientific literature remains predominantly focused on male athletes. This study aimed to identify the technical and tactical actions that distinguish winner from loser boxers in elite amateur women’s boxing by analysing the final matches of the 2023 IBA Women’s World Boxing Championships. **Methods**: Twelve final bouts (one per weight-category) were analysed using a customised notational framework based on the offensive–defensive interaction cycle. A total of 1323 offensive and 1456 defensive actions were recorded and categorised using Kinovea (0.9.5 release) software and an Excel dashboard. Offensive Effectiveness Ratio (OER) and Defensive Effectiveness Ratio (DER) were calculated, and Chi-square tests with standardised residuals were applied to detect associations between action types and bout outcomes. **Results:** Winners performed fewer offensive actions (n = 635) than losers (n = 688) but demonstrated significantly higher OER across all rounds (W: 0.39-0.38-0.39; L: 0.26-0.28-0.29). Winners landed more punches to the head and made greater use of rear-hand hooks and uppercuts. Pivoting and shifting back, particularly when followed by counterattacks, were significantly associated with winners (*p* < 0.001). Conversely, parrying and single counterpunches were linked to losing outcomes. DER values suggested superior defensive efficiency among winners. **Conclusions**: Winner boxers showed superior technical effectiveness, selective use of single and rear-hand punches and used more dynamic defensive strategies. These findings support using notational analysis in high-performance coaching and emphasise the need for more research on elite female combat athletes.

## 1. Introduction

Although evidence indicates that fighting exhibitions can be traced to ancient civilisations such as Mesopotamia, Egypt, Minoan Crete, Greece, and Rome [1], and that boxing was first introduced during the 23rd Ancient Olympic Games in 688 BCE, it differed significantly from the modern sport as it is known today. In its early days as a modern sport, boxing rules were poorly defined and varied from one exhibition to another. In 1743, the first set of seven rules, known as the “Broughton’s Rules” was formulated. According to these rules, if a man went down and could not continue after a count of 30 s, the fight was over. After modifications in 1843 and 1853, the early rules were replaced in 1867 by the twelve “Marquess of Queensberry Rules”. These rules are considered the foundation of modern boxing and introduced key regulations such as a 24-foot-square ring, three-minute rounds with one-minute rest intervals, a ten-second count for knockdowns, the use of gloves and the prohibition of wrestling. The modernization of boxing continued throughout the 20th century, with the establishment of governing bodies and formalised weight classes. In 2013, the Amateur International Boxing Association (AIBA) implemented several substantial changes to the rules of elite male amateur boxing [2]. Notably, headguards were removed from men’s amateur boxing, although they remained. The AIBA also introduced four criteria for judges to determine the winner: (i) the number of quality punches landed in the target area, (ii) domination of the bout by technical and tactical superiority, (iii) competitiveness and (iv) infringement of the rules.

The type and quantity of punches, punch combinations, movement around the ring, and activity rates have all been influenced by previous changes to boxing rules and structure. For instance, before 2013, boxers often relied on short-range techniques to stay close to their opponents. After 2013, they began using more foot movement to engage and retreat, incorporating long-range punching techniques, and making deliberate defensive movements to avoid opponents’ punches. Additionally, there has been heightened concern about vulnerability to knockouts due to the removal of headguards [2]. Boxing has also undergone significant transformation in terms of gender inclusivity, particularly with the inclusion of women’s boxing in the Olympic Games. This shifting began at the 2012 London Olympics, where women competed in three weight categories: flyweight, lightweight, and middleweight. By the Paris 2024 Olympics the number of categories has expanded to six: flyweight, bantamweight, featherweight, lightweight, welterweight, and middleweight [3]. Additionally, a significant change in the women’s competition format has increased the bout duration from four 2-min rounds to three 3-min rounds, aligning with the men’s competition format [4]. These changes reflect the International Olympic Committee’s (IOC) ongoing commitment to gender equality and greater inclusivity in sport. This is further emphasised in the IOC Portrayal Guidelines, which promote fair, balanced, and non-stereotypical representation of athletes across all genders in sports media and communication [3].

The scoring system used in Olympic boxing is based on the Ten Point Must System, where judges assign 10 points to the winner of each round, and between 7 and 9 points to the loser, depending on the degree of superiority demonstrated during the round. This system ensures a clear determination of the winner in each round [4]. This evolution, from a scoring system focused solely on punch volume to one that emphasises punch effectiveness, has significantly influenced the athletes’ tactical approaches to the match.

The impact of these changes on performance is reflected by the growing body of literature on performance analysis, which quantifies key factors contributing to success in boxing [5]. Notational analysis has proven to be a valuable tool for performance evaluation across various combat sports [6,7,8,9]. However, despite the significant global growth of boxing, particularly among women, its application remains limited [10]. Although female participation in major boxing events such as the Olympics has increased, there is still a noticeable lack of studies focused specifically on female boxers. Most existing research predominantly focuses on male athletes, creating a gap in understanding the performance indicators and tactical strategies unique to women’s boxing [11].

Specifically, a study by Thomson et al. [10] analysed male boxers in 3 × 2-min matches and developed a framework of 25 performance indicators, including offensive, defensive, and counter-offensive actions, which proved valuable for providing a consistent and systematic approach to boxing performance analysis. Davis et al. [12] reported that Olympic level male boxers maintained an activity rate of 1.4 actions per second (20 punches, 2.5 defensive movements, and 47 vertical hip movements per minute), with winners showing greater punching accuracy, particularly in the later rounds, compared to losers. Similarly, Wandee and Benjapalakorn [13], analysing winning male boxers at the 2012 Olympic Games, highlighted the predominance of straight punches and combinations, together with higher accuracy and activity rates in the final rounds, underscoring the technical and tactical demands required for success at the elite level. In a study on Egyptian male boxers, El Ashker [14] reported that straight punches were the most frequently used technique, primarily targeting the head, while uppercuts were the least commonly executed. This finding also aligns with the results of Kapo et al. [15] on elite male amateur boxers, regarding straight punches, although they also found that winners more effectively used hooks, uppercuts, and combined and defensive techniques compared to losers. El Ashker’s [14] study also found that defensive skills were similar between winners and losers, with successful boxers employing comparable offensive and defensive strategies. In contrast, a study by Shtanagei [11] on female boxers during the 2023 European Under-22 Championships revealed that winning athletes delivered 8.4% more punches than their opponents, particularly in the third round. Interestingly, Shtanagei’s findings [11] showed a preference for uppercuts among female boxers, which contrasts with previous research on elite male counterparts [14,15]. Additionally, Davis [16], in his comparative analysis of female and male boxers, identified key factors that distinguish winning female boxers from their losing counterparts. Successful female boxers demonstrated advanced skills in using the rear hand for straight punches, executing body uppercuts, employing superior defensive techniques, and maintaining a lower percentage of missed punches.

Considering the changes in competition formats and the evolution of performances, it is essential to further explore the specific techniques and performance indicators that characterise female boxing. Therefore, this study aimed to analyse performance characteristics in elite amateur women’s boxing by examining video footage from the 2023 IBA Women’s World Boxing Championships held in New Delhi. The primary objective was to identify the technical and tactical elements that differentiate winning (W) from losing (L) athletes throughout the course of the bout. The analysis was based on the concept of the interaction cycle between athletes, understood as the continuous and reciprocal exchange of offensive and defensive actions in response to the opponent’s behaviour. The findings are intended to provide evidence-based insights to support coaching strategies and promote performance development in high-level women’s boxing.

## 2. Materials and Methods

### 2.1. Experimental Design

**Figure 1:**
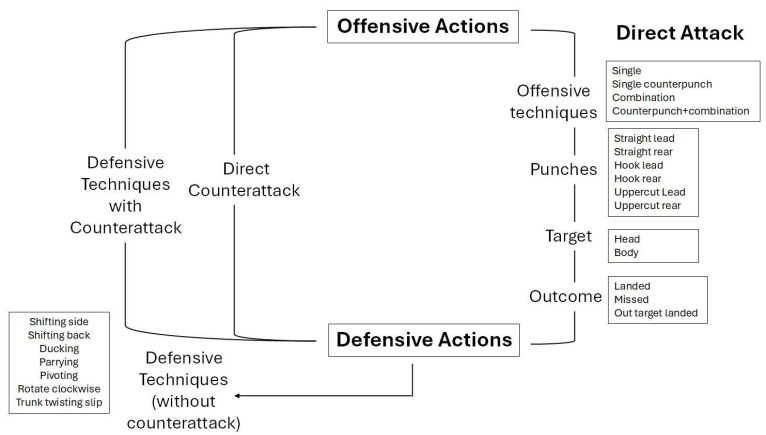
Boxing interaction cycle.

Since, during a match, the two boxers are engaged in continuous technical and tactical exchanges—such as throwing punches, dodging, feinting, and parrying—these actions form a dynamic cycle in which each boxer’s response provokes and influences the other’s [17]. For this reason, the present analysis adopted a model centred on the interaction cycle between boxers (Figure 1), in line with previous attempts to develop structured frameworks for identifying performance priorities in individual sports [18]. The actions were categorised within a circular framework that distinguishes offensive and defensive behaviours. Table 1 outlines the framework used to analyse offensive and defensive actions, detailing the types of attacks, specific punches or combinations executed, targeted areas and their respective outcomes. It also describes the types of defensive actions and techniques employed. Each defensive action was classified as either a standalone evasion (i.e., defensive techniques without counterattack), a compound action involving an immediate counterattack (i.e., defensive techniques with counterattack) or as an offensive action performed during a defensive phase (i.e., direct counterattack). Thus, a technical profile of the 12 final matches of the 2023 IBA Women’s World Boxing Championships was analysed according to the fighting phase (F), which is defined as the moments when the two boxers exit the safety distance and enter the fighting distance, engaging in active technical and tactical exchanges such as attacks, defences, and counterattacks. This phase was further subdivided into two sub-phases: punch (P), referring to technical exchanges of punches, and clinch (C), referring to situations in which one or both boxers engage in close-range combat, resulting in a momentary locking of their arms [17]. According to the 2023 IBA rules, all matches were fixed in 3 rounds (1st, 2nd and 3rd) of 3 min, with one minute of rest in between, for all 12 weight categories (i.e., 48 kg, 50 kg, 52 kg, 54 kg, 57 kg, 60 kg, 63 kg, 66 kg, 70 kg, 75 kg, 81 kg, and 81+ kg) [4].

### 2.2. Procedures

All matches’ recordings were downloaded by the YouTube IBA Channel and downloaded in HD resolution (1080 p, 50 fps) [19], for this reason, since free public access, no informed consent was required.

The frequency of occurrence of all number of offensive and defensive actions, the number of attack types and different types of defensive action for match outcome (i.e., winners and losers) was counted and annotated by means of a customised Excel dashboard (Microsoft Office 365, setup.exe version 16.0) using a free video annotation tool designed for motion analysis (Kinovea©, 0.9.5 release), with speed adjustable in 0.1 s, which allowed the slow-motion replay as well as rewinding the contest and watching events frame-by-frame. The same researcher, qualified as a boxing coach by the Italian Boxing Federation, analysed footage of all matches and all rounds [17]. The analyses were provided twice, four weeks apart, by the same researcher, and subjected to intra-observer reliability analysis (ICC = 1.0).

### 2.3. Statistical Analysis

Given the nominal data of this study, a Chi-square test of independence was performed to examine the association between the offensive and defensive actions and match outcome (i.e., winners or losers). Post hoc analyses for the Chi-square test were performed using standardised residuals with a threshold of Z > 1.96. Standardised residuals were calculated using IBM SPSS Statistics ver. 25.0 (IBM Co., Armonk, NY, USA), according to the procedures described by Field [20]. In line with previous approaches to performance indicators in boxing [14], who proposed measures of offensive and defensive effectiveness, we developed two customised indices: the Offensive Effectiveness Ratio (OER) and the Defensive Effectiveness Ratio (DER).

The OER was defined as the number of punches landed divided by the total punches thrown by each boxer. The landed punches included those directed to the head and/or body that successfully hit the target areas permitted by the regulations.

The DER was defined as the number of boxers’ defensive actions divided by the total number of opponent’s missed punches. The DER quantifies the effectiveness of defensive actions in neutralising the opponent’s attacks and reflects the proportion of successful defensive manoeuvres.

Both OER and DER were calculated for each round with data pooled for weight categories.

## 3. Results

During the twelve finals, boxers executed a total of 1323 offensive actions and 1456 defensive actions. Table 2 shows the frequencies of occurrence of offensive and defensive actions across the 12 weight categories.

Data pooled for match outcomes indicated that winner boxers performed fewer offensive (N = 635 vs. N = 688) and more defensive (N = 808 vs. N = 648) actions compared to loser boxers, respectively. Table 3 presents the frequency of occurrence (%) of offensive and defensive action components, respectively.

The Chi-square test showed a significant association between the type of offensive action and match outcome (χ^2^ (3) = 13.4, *p* = 0.004). The post hoc test showed that single offensive actions were significantly associated with winners (residual = +2.98), while counterpunch+ combination actions were marginally associated with loser boxers (residual = +1.96). No significant differences were observed for combination or single counterpunch actions, as their residuals did not exceed the ±1.96 threshold.

The Chi-square test showed a significant association between the type of defensive action and match outcome (χ^2^ (17) = 83.2, *p* < 0.001). Post hoc analysis revealed that pivoting (standardised residual = +4.77) and shifting back (standardised residual = +2.99) were significantly associated with winners, while pivoting + counterattack (standardised residual = +2.20) also showed a significant positive association with victory. Although shifting back + counterattack displayed a positive trend for winners (residual = +1.79), it did not reach the statistical significance threshold (Z > 1.96). On the contrary, parrying (standardised residual = +4.61) and parrying + counterattack (standardised residual = +3.68) were significantly associated with loser boxers. Additionally, single counterpunches were marginally associated with losing outcomes (residual = +1.98). No other defensive actions exceeded the ±1.96 threshold for statistical significance.

Regarding the total number of punches delivered during both the offensive and defensive actions, data pooled for weight categories showed a similar count for winner (n = 1951) and loser (n = 1930) boxers. The descriptive statistics of the types of punch and the used hand, in relation to winners and losers and divided per rounds are depicted in Table 4. The most frequently punches delivered by both winners and losers were the straight punches executed with the lead hand.

Table 5 shows the count of landed, missed, and out-of-target punches, pooled for weight categories, delivered to both head and body in relation to match outcome (winner vs. loser) and round (1st, 2nd, and 3rd).

Table 6 shows the Offensive (OER) and Defensive (DER) Effectiveness Ratio for both winners and losers boxers, analysed across the three rounds of competition.

## 4. Discussion

This study represents the first comprehensive notational analysis of the Elite Women’s World Boxing Championship in the 3 × 3-min-bout format. It investigates the types, frequency, and outcomes of boxers’ technical exchanges during the fighting phase, with the goal of identifying the technical and tactical profiles that distinguish winners from losers in elite women’s amateur boxing.

The main findings of this study are as follows: (a) Winner boxers executed fewer offensive actions than their losers counterpart but demonstrated a greater overall efficiency, as evidenced by consistently higher Offensive Effectiveness Ratios (OER). (b) Straight punches, delivered with both the lead and rear hands, were the most frequently employed offensive techniques among both winners and losers. (c) Loser boxers tended to throw a higher number of straight punches, particularly with the rear hand; however, these were characterised by lower execution effectiveness, as indicated by lower OER values and fewer punches landing on the head. (d) Winner boxers consistently landed a significantly greater number of head punches across all three rounds. (e) Although winner boxers initially executed fewer missed body punches, both groups showed a comparable increase in missed attempts in the third round, suggesting a general decline in accuracy due to fatigue rather than a technical disparity. (f) Chi-square analysis revealed a significant association between the use of single punches and successful bout outcomes. (g) Rear-hand hooks and uppercuts were more frequently employed by winner boxers. (h) Defensive actions such as pivoting and shifting back were significantly more common among winners, enabling better control of the bout and minimising opponent scoring opportunities, while more static defensive techniques, such as parrying, were associated with loser boxers. (i) Overall, superior technical effectiveness, both offensive and defensive, emerged as critical determinant of success in elite-level women’s amateur boxing.

In our study, winner boxers performed fewer offensive actions compared to losers, suggesting a more selective and strategic approach. This fact is further supported by the higher number of single punches rather than combination punches thrown by winners, whereas losers preferred combinations over single punches. Concerning the total number of punches, our study showed that winners threw 1.1% more punches than losers. This trend aligns with a study by Shtanagei et al. [11], which analysed the same number of matches (=12) during the U22 European Championship, and reported an 8.4% increase in punches by winners. Notably, in our study, pooled data for weight categories showed that first and third round were the rounds had the highest scores for winners and losers, respectively, (1st round: 35.6% vs. 33.2%; 2nd round: 30.9% vs. 31.7%; 3rd round: 33.4% vs. 35.1%). This pattern differs from Shtanagei et al. [11], who observed that winners delivered more punches in the third round. These differences may be attributed to variations in athletes’ experience and the higher technical demands of female boxing at the World Championship level, which often necessitate a more tactical approach, especially in the (third) concluding round.

Regarding the type of punches, the straight punches, executed with both the lead and rear hand were the most frequent attacking techniques for both winner and losers. This finding aligns with El Ashker’s [14] study analysing technical and tactical aspects in a national male boxing championship. Specifically, our study on female boxing, showed that losers threw a higher volume of straight punches—both with lead hand (W: n = 640; L: n = 756) and rear hand (W: n = 445; L: n = 510). This fact underlines that, although losers made the most extensive use of f straight punches, their higher volume suggests potential issues with accuracy and effectiveness. Indeed, winners demonstrated a greater accuracy in landing punches on the opponent’s head across all rounds (1st round: W = 221, L = 141; 2nd round: W = 189, L = 143; 3rd round: W = 213, L = 145). In contrast, the number of punches successfully landed on the body was slightly lower for winners in the third round (1st round: W = 48, L = 41; 2nd round: W = 39, L = 38; 3rd round: W = 39, L = 53). These data on landed punches are in line with those on missed, which showed a higher number of total missed punches on head and body across all rounds (1st round: n = 372; 2nd round: n = 380; 3rd round: 429) for losers. Notably, losers recorded twice as many missed body punches as winners (L = 46; W = 23), indicating a lower accuracy in the first round. In the second round, both groups showed a reduction in missed body punches (L = 20; W = 12), with winners demonstrating a notably lower error rate. However, in the third round, the missed body punches increased for both winners and losers (W = 31; L = 31), reflecting a potential boxers’ fatigue status, consistent with fatigue-related adjustments in motor control and coordination [21], and a shift toward more aggressive tactical behaviours.

Overall, these findings on landed and missed punches suggest that winner boxers were more selective in their targeting, primarily focusing on the head punches, which are more likely to influence judges’ scoring due to their visible impact, such as head retraction or neck movement. Conversely, missed body punches alone do not appear to effectively differentiate winners from losers but may instead reflect broader tactical and physiological dynamics that fluctuate across rounds.

The chi-square analyses revealed significant associations between both offensive (χ^2^ (3) = 13.3, *p* = 0.004) and defensive action types (χ^2^ (17) = 83.2, *p* < 0.001) and the match outcome, indicating that certain technical–tactical behaviours could be linked to successful or unsuccessful performance. In particular, the post hoc analysis of standardised residuals showed that the execution of single offensive actions was significantly associated with winner boxers (residual = +2.98), suggesting that simpler, direct attacks may be more effective under competitive pressure. While counterpunch + combination actions were marginally associated with loser boxers (residual = +1.96). suggests that clear, well-executed, and direct punches, likely more visible and easier to evaluate by judges, are more effective in scoring and influencing bout outcomes. This finding aligns with the idea that, in high stakes matches such as world championship finals, athletes who prioritise precision over volume or complexity may gain a strategic advantage.

Winner boxers also demonstrated greater use of lead-hand hooks (W: n = 465; L: n = 399), while rear-hand hooks, though less frequent overall, were still more common among winners (W = 12.3%, L = 7.9%). Although uppercuts were the least used punch type, contrary to the findings of El Ashker’s [14] findings, winner boxers demonstrated superior execution with rear-hand uppercuts (n = 136 vs. L: n = 88). These results suggest that, despite their lower frequency, rear-hand hooks and uppercuts may represent strategic tools in elite women’s boxing. Further supporting the earlier findings regarding offensive actions, the data in Table 4 shows a noticeable decrease in lead straight punches among winner boxers across the rounds, while losers demonstrated an increase in their lead straight punches from the second to the third round. This dynamic change in punching strategy highlights differences in tactical approaches as the match progresses. Moreover, winners consistently landed a higher proportion of hook punches (12.3%, n = 240) compared to losers (7.9%, n = 153), suggesting that hooks, especially rear-hand hooks, were more effectively utilised by winners as part of their strategic repertoire. The increased frequency of successful rear-hand uppercuts (W: n = 136 vs. L: n = 88) further reinforces the importance of technical variety and precision in elite boxing. These observations indicate that, despite their lower frequency, the effective use of hooks and uppercuts played a significant role in providing winners with a tactical advantage.

The most frequently adopted defensive techniques by winners were shifting back (W: 23.3%; L: 16.8%), an effective strategy for enabling rapid counterattacks. Winner boxers combined this movement with counterattacks more frequently (14.9%) than losers (11.7%). Regarding the direct counterattacks, the winners used most frequently the counterpunch+ combination (19.4%) while the losers performed most the single counterpunch (21.6%). In contrast, parrying techniques, both with and without counterattacks, were more commonly employed by losers compared to winners (14.1% vs. 4.8%). Pivoting, which is a more dynamic repositioning strategy, was rarely used by losers (0.9%) but was notably more frequent among winners (5.6%). These results suggest that winners tend to adopt a more proactive and mobile defensive style, favouring evasive actions that allow them to avoid punches altogether and reposition effectively. In contrast, static defences such as parrying appear less effective in elite women boxing, potentially due to their limited ability to neutralise the opponent’s initiative or influence judges’ scoring. The chi-square analysis confirmed that pivoting (standardised residual = +4.77) and shifting back (standardised residual = +3.31) were significantly associated with victory, whereas parrying (standardised residual = +4.61) and parrying + counterattack (standardised residual = +2.19) were associated with defeat. Moreover, the single punch as a direct counterattack, was significantly associated with losers’ outcomes (standardised residual = +2.05), suggesting that isolated punches thrown in response to an attack—without being part of a more structured combination or counterstrategy, may be less effective in high-level competitive contexts. This highlights the tactical importance of dynamic movement in high-level performance. No other defensive actions exceeded the ±1.96 threshold for statistical significance.

These findings diverge from those of Davis et al. [22], who observed that fewer defensive movements were associated with victory in male boxers, implying a different interpretation or valuation of defensive behaviours across genders. Moreover, Davis et al. [16] reported that female winners employed significantly more foot-based defences under a 4 × 2-min-bout format, particularly in the final round, which support the present study’s emphasis on evasive strategies.

While parrying remains a fundamental defensive skill, excessive reliance on it without subsequent follow-up action may be perceived as passive, potentially affecting judges’ perceptions. Additionally, parrying may not always be clearly visible to all judges, and because it involves physical interaction, it can be mistakenly interpreted as a landed punch, thereby adversely affecting the evaluation. In contrast, dynamic defensive actions, such as pivoting and shifting back, may better reflect active bout control and are thus more favourably judged. Moreover, these findings suggest that judges may favour clear, well-executed actions—such as clean single punches or effective evasive movements—over disorganised or ambiguous exchanges, reinforcing the importance of precision and tactical clarity in achieving a winning outcome.

Regarding the OER and DER, despite some methodological differences in the formulas used across the two studies, our findings align with those of El Ashker [14] emphasising the role of technical effectiveness in determining success in competitive boxing. In our study, winners consistently exhibited higher OER values across all rounds compared to losers, indicating a superior offensive efficiency (Table 6). Conversely, DER data showed greater losers’ defensive effectiveness in the first round compared to winners (69% vs. 58%), while the latter displayed progressive higher values across the subsequent rounds, reaching 70% in the third round (Table 6). This pattern suggests a higher winners’ efficiency to neutralise opponents’ attacks as the bout progressed, demonstrating superior timing and tactical abilities to effectively manage rounds, as well as physical conditioning. Consistent with previous research on male boxers [14], our results show that also in elite women amateur boxing success is closely linked to technical and tactical efficiency, and dynamic defensive abilities.

These insights reinforce the critical importance of maintaining technical and tactical efficiency throughout the entire match, particularly as fatigue sets in. Incorporating detailed notational analysis into technical and tactical training programmes can provide coaches with valuable tools to optimise performance in elite women’s boxing, enabling targeted improvements in strategy, technique, and fatigue management.

A limitation of this study is the small sample size, which only includes the boxer finalists of the 12 weight-category finals of the 2023 Women’s Boxing World Championships. Although these athletes represent a very high worldwide level of women’s boxing competition, the limited number of matches does not allow for robust inferential analyses or post hoc statistical power calculations, in line with the exploratory and descriptive nature of the study.

Furthermore, all observations were conducted by a single analyst; although intra-observer reliability was high, involving multiple observers in future studies could further strengthen the robustness of the results.

Future research should also aim to expand sample sizes, including athletes from different competitive levels, and investigate how dynamic and proactive defensive strategies contribute to successful performance outcomes and explore technical–tactical patterns using multiple comparisons in hypothesis-driven studies.

## 5. Conclusions

This study provides a detailed notational analysis of the final matches of the 2023 IBA Women’s World Boxing Championships, offering new insights into the technical and tactical behaviours that differentiate winning athletes from their losing counterparts in the 3 × 3-min-bout format. These results highlight that successful boxers adopt a more selective offensive strategy, favouring simple and well-timed actions, such as single punches, over a high volume of actions performed by combinations. Notably, although overall less frequent, rear-hand hooks and uppercuts proved to be effective scoring punches, suggesting that possessing a broad variety of techniques can provide a tactical advantage for female boxers.

From a defensive perspective, winners demonstrated a clear preference for dynamic defensive techniques, particularly in pivoting and shifting back movements, as opposed to more static techniques such as parrying, which was more frequently associated with losers. These mobile defences not only allow boxers to evade opponents’ punches but also create opportunities for effective counterattacks. Moreover, the combination of defensive techniques and immediate counterattacks proved to be a decisive tactical resource in high-level women boxing. From an applied standpoint, coaches working with elite female boxers are encouraged to: (a) prioritise quality over quantity in offensive actions, promoting accurate and well-targeted punches, especially to the head and with the rear hand; (b) incorporate rear-hand hooks and uppercuts into the technical repertoire, training athletes to execute them from favourable positions and within structured combinations; (c) enhance dynamic defensive skills, such as pivoting and shifting back or shifting side techniques, with a focus on rapid repositioning and readiness to counterattack; (d) avoid an over-reliance on parries, as they may not be clearly perceived by judges whom, due to their physical interaction, can sometimes mistake them for a landed punch by the opponent, potentially leading to a misjudgement in scoring if not followed by an assertive counteraction; (e) design drills that simulate fatigue conditions to maintain technical efficiency and decision-making clarity throughout the entire progression of the match.

Integrating these findings into technical–tactical training programmes can help coaches better align female boxers’ strategies with the key performance indicators observed in top-level matches. A data-driven approach can facilitate more effective and targeted coaching interventions, ultimately enhancing performance and success in elite international women’s boxing.

## Figures and Tables

**Table 1:** Offensive and Defensive Phases Framework.

Actions	
Offensive Actions	Any boxer’s intentional attempt to hit the opponent by moving from a safe to a fighting distance while throwing a single punch or a combination of punches with either the lead or rear hand, using straight, hook, or uppercut techniques.
Defensive Actions	Any attempt to avoid a direct attack through (a) a defensive technique without counterattacking, with the goal of maintaining a safe distance; (b) executing a defensive technique followed by a counterattack; or (c) responding with a direct counterattack.
Techniques	
Offensive Techniques	
Single Punch	An action with a single punch.
Single Counterpunch	An action with a single counterpunch combined with a forward step.
Combination	An action with more than one punch.
Counterpunch + Combination	An action with more than one punch. The combination always begins with a counterpunch combined with a forward step.
Defensive Techniques	
Shifting Side	Lateral movement or semi-lateral movement where on or both feet move sideways, maintaining a guard position to avoid a punch.
Shifting Back	Backward or semi-backward movement where one or both feet retreat, distancing the boxer from the opponent to avoid a punch.
Rotate Clockwise	Circular slip involving the head, trunk, and feet to evade a punch.
Ducking	Knee flexion to lower the body and reduce the target area.
Trunk Twisting Slip	A slip combining trunk flexion and rotation to avoid a punch.
Parrying	A movement where the boxer uses the arms to cover the target area, deflecting the opponent’s punch.
Pivoting	A movement where the boxer fixes the lead foot on the ground and rotates, shifting weight to the opposite foot to evade a punch.
Punches	
Straight Lead	A straight punch from the lead hand moving along the sagittal plane.
Straight Rear	A straight punch from the rear hand moving along the sagittal plane.
Hook Lead	A punch from the lead hand moving along the transverse axis in a lateral motion.
Hook Rear	A punch from the rear hand moving along the transverse axis in a lateral motion.
Uppercut Lead	A punch from the lead hand moving along the sagittal plane and longitudinal axis, starting with a downward projection and ending with an upward projection.
Uppercut Rear	A punch from the rear hand moving along the sagittal plane and longitudinal axis, starting with a downward projection and ending with an upward projection.
Target + Outcome	
Landed Head	A punch landed to the head if it visibly hits the opponent’s face (target area permitted by the rules).
Missed Head	A punch is labelled as missed to the head if it fails to hit the opponent’s face (target area permitted by the rules).
Landed Body	A punch is labelled as landed to the body if it visibly hits the opponent’s front torso above the belt (target area permitted by the rules).
Missed Body	A punch is labelled as missed to the body if it fails to hit the opponent’s front torso above the belt (target area permitted by the rules).
Out Target Landed	A punch is labelled as out target when it hits the opponent with visible impact but outside the target area permitted by the rules.

**Table 2:** Frequencies of occurrence of offensive and defensive actions across different weight categories.

		Offensive Actions (n+%)	Defensive Actions (n+%)	Total Actions (n)
Weight categories	45–48 kg	116 (51.1)	111 (48.9)	227
	48–50 kg	60 (51.3)	57 (48.7)	117
	50–52 kg	111 (52.9)	99 (47.1)	210
	52–54 kg	156 (47.7)	171 (52.3)	327
	54–57 kg	69 (44.5)	86 (55.5)	155
	57–60 kg	83 (43.5)	108 (56.5)	191
	60–63 kg	159 (45.6)	190 (54.4)	349
	63–66 kg	108 (48.2)	116 (51.8)	224
	66–70 kg	152 (48.6)	161 (51.4)	313
	70–75 kg	123 (46.1)	144 (53.9)	267
	75–81 kg	68 (45.3)	82 (54.7)	150
	81+ kg	118 (47.4)	131 (52.6)	249

**Table 3:** Frequency of occurrence of offensive and defensive techniques.

	Winners	Losers
Offensive techniques		
Single	49.5	41.3
Single Counterpunch	3.0	4.9
Combination	46.6	51.5
Counterpunch + Combination	0.9	2.3
	100	100
Defensive Techniques		
Single	1.7	3.4
Single Counterpunch	17.7	21.6
Combination	1.6	1.5
Counterpunch + Combination	19.4	17.1
Shifting Side	0.9	1.5
Shifting Back	23.3	16.8
Rotate Clockwise	1.7	1.5
Ducking	4.0	5.4
Trunk Twisting Slip	0.5	0.5
Parrying	3.6	9.7
Pivoting	5.6	0.9
Shifting Side + Counterattack	0.7	1.4
Shifting Back + Counterattack	14.9	11.7
Rotate Clockwise + Counterattack	0.7	0.8
Ducking + Counterattack	1.1	0.9
Trunk Twisting Slip + Counterattack	0.7	0.9
Parrying + Counterattack	1.2	4.4
Pivoting + Counterattack	0.7	0
	100	100

**Table 4:** Count and frequency of occurrence of the different types of punches between winners and losers across each round.

Types of Punches	Counts (n)	Percentage (%)	Hand	Outcome	Round 1	Round 2	Round 3
Straight	640	32.8	Lead	Winners	224	211	205
	756	39.2		Losers	271	235	250
	445	22.8	Rear	Winners	161	142	142
	510	26.4		Losers	150	169	191
Hook	465	23.8	Lead	Winners	157	137	171
	399	20.7		Losers	133	133	133
	240	12.3	Rear	Winners	90	63	87
	153	7.9		Losers	49	46	58
Uppercut	25	1.3	Lead	Winners	9	9	7
	24	1.2		Losers	8	7	9
	136	7.0	Rear	Winners	54	41	41
	88	4.6		Losers	30	22	36
Total	1951	100		Winners	695	603	653
	1930	100		Losers	641	612	677

**Table 5:** Count of landed, missed, and out-of-target punches to head and body, by winner and losers across each round.

Round	Outcome	Landed Head	Missed Head	Landed Body	Missed Body	Out Target Landed
1st	Winners	221	349	48	23	61
	Losers	141	383	41	46	46
2nd	Winners	189	311	39	12	52
	Losers	143	360	38	20	59
3rd	Winners	213	332	39	31	63
	Losers	145	398	53	31	50

**Table 6:** Offensive (OER) and Defensive Effectiveness Ratios (DER) across rounds.

Technical Performance Ratios	Outcome	Round 1	Round 2	Round 3
Offensive Effectiveness Ratio	Winners	0.39	0.38	0.39
	Losers	0.26	0.28	0.29
Defensive Effectiveness Ratio	Winners	0.58	0.67	0.70
	Losers	0.69	0.59	0.60

## Data Availability

Data are contained within the article.

## Abbreviations

The following abbreviations are used in this manuscript:

AIBA	Amateur International Boxing Association
IOC	International Olympic Committee
IBA	International Boxing Association
W	Winners
L	Losers
F	Fighting phase
P	Punch
C	Clinch
OER	Offensive Effectiveness Ratio
DER	Defensive Effectiveness Ratio
